# Bioactive Components, Pharmacological Properties, and Applications of *Cistanche deserticola* Y. C. Ma: A Comprehensive Review

**DOI:** 10.3390/nu17091501

**Published:** 2025-04-29

**Authors:** Xiaofeng Liu, Zichao Yang, Minjun Han, Yao Zhang, Hussain Muhammad, Hao Zhong, Rongfa Guan

**Affiliations:** 1 College of Food Science and Technology, Zhejiang University of Technology, Hangzhou 310014, China; lxf0817@zjut.edu.cn (X.L.); 221123260067@zjut.edu.cn (Z.Y.); 221124260073@zjut.edu.cn (M.H.); mhussainft11@gmail.com (H.M.); zhonghao@zjut.edu.cn (H.Z.); 2 Zhejiang Provincial Key Laboratory for Chem and Bio Processing Technology of Farm Produces, School of Biological and Chemical Engineering, Zhejiang University of Science and Technology, Hangzhou 310023, China

**Keywords:** *Cistanche deserticola* Y. C. Ma, bioactive compounds, antifatigue, Immune regulation, application

## Abstract

*Cistanche deserticola* Y. C. Ma (*C. deserticola*), a holoparasitic desert plant traditionally revered as “desert ginseng”, has emerged as a versatile resource with significant applications in both medicinal and dietary contexts. This comprehensive review systematically explores its bioactive constituents, including phenylethanol glycosides (PhGs), iridoids, lignans, and polysaccharides, and elucidates their multifaceted pharmacological properties. Contemporary research substantiates the therapeutic potential of *C. deserticola*, demonstrating its anti-inflammatory, antioxidant, antitumor, neuroprotective, and immunomodulatory effects. Mechanism analysis elucidated its anti-fatigue and immune-enhancing activities, primarily through the modulation of pivotal signaling pathways, including NF-κB, AMPK, and TLR4. The review also highlights recent regulatory advancements in China, which have approved *C. deserticola* as a functional food ingredient, complementing its traditional uses in kidney yang tonification and promoting intestinal health. Despite its promising attributes, challenges related to sustainable cultivation and clinical application remain. By integrating ethnopharmacological wisdom with modern scientific evidence, this work lays a robust foundation for advancing the applications of *C. deserticola* in nutraceuticals and therapeutics.

## 1. Introduction

*Cistanche deserticola* Y. C. Ma (*C. deserticola*), commonly known as “desert ginseng”, is a holoparasitic herbaceous perennial belonging to the Orobanchaceae family. Adapted to arid ecosystems, it survives by forming specialized haustoria to extract water and nutrients from host plants, primarily *Tamarix* spp. Its morphological features include fleshy yellow stems and reduced scale-like leaves, typically found in desert regions of Inner Mongolia, Gansu, and Xinjiang in China. Historically documented in early pharmacopoeias, including “Compendium of Materia Medica”, *C. deserticola* has long been renowned for its ability to tonify the liver and kidneys, nourish essence and blood, and strengthen muscles and bones.

In recent years, *C. deserticola* has gained recognition not only as a medicinal herb but also as a safe and functional food ingredient. In 2016, the China National Center for Food Safety Risk Assessment (CFSA) evaluated *C. deserticola* from the Alxa Desert and confirmed its safety for human consumption based on toxicological and safety data. By 2018, it was included in the “List of Substances that Are Both Food and Traditional Chinese Medicine” by the National Health Commission, affirming its suitability for daily dietary use. In 2020, *C. deserticola* was officially approved as a dual-purpose substance for both medicinal and food applications, following a pilot program initiated by the National Health Commission and the State Administration for Market Regulation. This regulatory approval has facilitated its integration into the food industry, with pilot programs for *C. deserticola*-based food products and raw materials launched in regions such as Inner Mongolia, Ningxia, Gansu, and Qinghai. As of June 2022, 60 health food products containing *C. deserticola* or its extracts have been registered on the National Specialty Food Information Platform, highlighting its growing popularity in the health food sector.

*C. deserticola* thrives in harsh desert environments characterized by extreme temperatures, low precipitation, and sandy or saline soils. These conditions are typical of the arid and semi-arid regions of northwestern China, where *C. deserticola* is naturally distributed. The plant’s ability to survive in such extreme environments is a testament to its remarkable ecological adaptations.

Recent scientific investigations have unveiled the multifaceted pharmacological properties of *C. deserticola*. Its extracts regulate bone metabolism, reduce inflammation, combat oxidative stress, modulate immune responses, and protect the nervous system. For instance, Wang et al. [1] demonstrated its dual role in promoting bone formation and inhibiting bone resorption, suggesting potential applications in osteoporosis treatment. Additionally, its anti-inflammatory [2] and central nervous system depressant effects [3] highlight its therapeutic versatility. These effects are attributed to bioactive compounds such as phenylethanol glycosides (PhGs) and polysaccharides, known for their antioxidant [4], immunomodulatory [5], and cellular protective properties [6]. Toxicological studies [7] have confirmed its safety, supporting its dual application in medicinal and dietary contexts.

Despite significant progress in understanding its pharmacological effects, challenges remain in the development and utilization of *C. deserticola*. Its niche ecological requirements complicate harvesting and preservation, and the optimization of extraction and purification techniques for its active compounds is still an area of active research. Additionally, although extensive research has validated its pharmacological effects, a deeper understanding of its mechanisms and clinical applications is needed.

This review systematically explores the bioactive compounds of *C. deserticola* and their pharmacological effects, with a focus on immune regulation and antifatigue mechanisms. By synthesizing existing research, we aim to provide a theoretical foundation for its development in the food and pharmaceutical industries and outline future research directions. This work is intended to inspire further investigation into *C. deserticola*’s bioactive compounds, promoting their application in health industries and contributing to improved human well-being.

## 2. Methodology

Data were obtained by searching various scientific online databases, including Google Scholar (https://scholar.google.com/), Baidu Scholar (https://xueshu.baidu.com/), ScienceDirect (https://www.sciencedirect.com/), Web of Science (https://www.webofscience.com/), PubMed (http://pubmed.cn/), Wiley (https://www.wiley.com/en-cn), Nature (https://www.nature.com/), SpringerLink (https://link.springer.com/), and ACS Publications (http://pubs.acs.org) using keywords such as *Cistanche deserticola* Y. C. Ma, bioactive compounds, antifatigue, immune regulation, phenylethanol glycosides, iridoid, lignans, antioxidant, antitumor, and anti-inflammatory.

## 3. Bioactive Constituents of *C. deserticola*

The multifunctional therapeutic properties of *C. deserticola*, such as anti-inflammatory, antitumor, and antioxidant activities, are primarily mediated by its key bioactive compounds: iridoids, lignans, polysaccharides, and phenylethanol glycosides (PhGs). The pharmacological functions of these compounds are summarized in Table 1.

### 3.1. Phenylethanol Glycosides

Phenylethanol glycosides (PhGs) are a structurally distinct class of glycosides characterized by a phenylethanol aglycone core, consisting of a benzene ring linked to an ethyl chain with a terminal hydroxyl group. Glycosylation occurs via β-glycosidic bonds to one or more monosaccharide units, such as glucose or rhamnose. As the principal bioactive constituents of *C. deserticola*, PhGs have been extensively studied for their anti-inflammatory, antioxidant, and hepatoprotective properties. Modern extraction methods, such as ultrasound-assisted ethanol extraction, are commonly used to optimize PhGs recovery while maintaining structural integrity.

**Figure 1:**
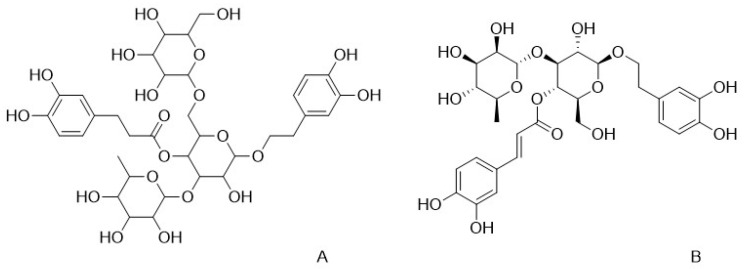
Chemical structures of echinacoside and acteoside, two key phenylethanol glycosides in *C. deserticola*. The image illustrates the molecular structures of echinacoside and acteoside. ((**A**), echinacoside; (**B**), acteoside).

To date, over 20 distinct PhGs have been isolated and characterized from *C. deserticola* (Table 2), each demonstrating unique therapeutic targets. In animal studies, echinacoside (Figure 1A) and acteoside (Figure 1B), two prominent PhGs, have been shown to protect against cerebral ischemia-reperfusion injury in mice, demonstrating dual hepatoprotective and neuroprotective effects [14,15]. In vitro experiments have revealed that tubuloside A modulates oxidative stress pathways to delay cellular senescence [16], while tubuloside B attenuates neuronal apoptosis through mitochondrial pathway regulation [17]. Acteoside provides cardioprotection by improving hypoxic tolerance and cerebral perfusion, thereby reducing the risk of vascular dementia [18]. In vitro studies have demonstrated that isoacteoside ameliorates renal tubular injury through inflammatory cytokine suppression [19], while 2′-acetylacteoside inhibits osteoclast differentiation via RANKL signaling downregulation, showing potential in osteoporosis management [20]. The structural diversity of PhGs supports their wide therapeutic applicability. Ongoing research is concentrated on understanding structure-activity relationships to inform and direct the development of targeted drugs.

Given the pharmacological significance of PhGs, particularly echinacoside and acteoside, efficient extraction and purification methods are essential. A standardized protocol involves grinding dried *C. deserticola* into powder and sieving to obtain a uniform sample. The powder is then mixed with an 80% ethanol-water solution at a mass ratio of 1:50 and subjected to ultrasonic-assisted extraction for 30 min to maximize compound release. After centrifugation to remove insoluble residues, the supernatant is collected and concentrated under reduced pressure using rotary evaporation to obtain a crude extract. Finally, high-speed counter-current chromatography (HSCCC) is employed for purification, leveraging the differential partition coefficients of PhGs in a two-phase solvent system to isolate echinacoside and acteoside with high purity and yield. HSCCC ensures the structural integrity and bioactivity of the target compounds, making it a preferred approach for large-scale preparation [21].

To accurately quantify the content of echinacoside and acteoside in the purified extracts, advanced analytical techniques are widely used. Ultra-performance liquid chromatography coupled with quadrupole-time-of-flight tandem mass spectrometry (UPLC-Q-TOF/MS) provides high sensitivity and specificity for identifying and quantifying these compounds based on their precise molecular weights and fragmentation patterns. Similarly, ultra-performance liquid chromatography with photodiode array detection (UPLC-PDA) allows for simultaneous detection and quantification of PhGs by analyzing their characteristic UV absorption spectra. High-performance liquid chromatography with electrospray ionization mass spectrometry (HPLC-ESI-MS) combines the separation power of HPLC with the detection sensitivity of mass spectrometry. This enables precise quantification of echinacoside and acteoside even at low concentrations. For routine analysis, high-performance liquid chromatography (HPLC) with UV detection remains a reliable and cost-effective method for determining the content of these bioactive compounds. These analytical techniques, as demonstrated in recent studies [3], ensure accurate quality control and facilitate the standardization of *C. deserticola* extracts for pharmacological applications.

### 3.2. Iridoid

Iridoids are a major class of bioactive compounds in *C. deserticola*, known for their antibacterial, anti-inflammatory, and hepatoprotective properties. To isolate these compounds, the dried material is typically extracted with an 85% ethanol-water solution, followed by sequential liquid-liquid partitioning using ethyl acetate and n-butanol. The iridoid-enriched fractions are then subjected to a multi-step purification protocol involving silica gel column chromatography, Sephadex LH-20 gel filtration, macroporous adsorption resin chromatography, reversed-phase ODS column chromatography, and semi-preparative HPLC. Final structural elucidation is achieved through combined analysis of MS and NMR spectra. Twenty-seven iridoids have been isolated from *C. deserticola*, all containing glucose monoglycosides. Typically, glucose is connected to the aglycone at the 1st position, while the 4th position may feature a carbonyl or demethylated group. Hydration occasionally occurs at the 3rd and 4th positions, and hydroxyl groups are commonly found at the 6th, 7th, 8th, or 10th positions. Dehydration at the 7th and 8th positions can form double bonds or epoxide ethers, while at the 10th, 1st, or 3rd positions, hydroxyl groups may dehydrate to form epoxide structures. The hydrogen atoms at positions 5 and 9 of the aglycone are in the β configuration.

**Figure 2:**
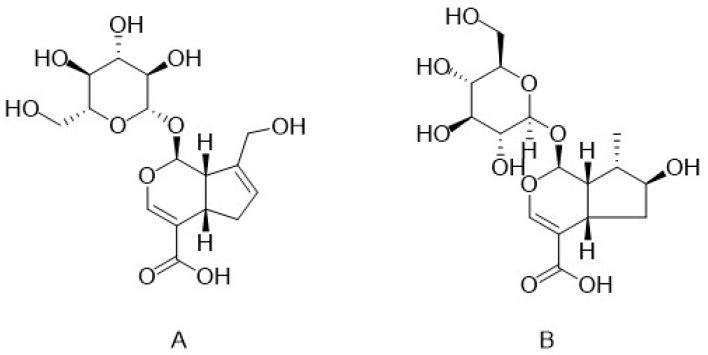
Chemical structures of geniposidic acid and 8-epiloganic acid in *C. deserticola*. The image illustrates the molecular structures of echinacoside and acteoside. ((**A**), geniposidic acid; (**B**), 8-epiloganic acid).

Cistanin, cistachlorin, bartsioside, 6-deoxycatalpol, geniposidic acid (Figure 2A), 8-epiloganic acid (Figure 2B), and gluroside are representative iridoids found in *C. deserticola*, which all have great anti-inflammatory activity [22,23]. Among them, 8-epiloganic acid not only possesses good anti-inflammatory effects but also shows strong antibacterial activity. In addition, geniposidic acid can enhance urine production and excretion [24].

### 3.3. Lignans

Lignans are naturally occurring phytochemicals composed of two phenylpropanoid units linked via carbon-carbon or carbon-oxygen bonds. In *C. deserticola*, these compounds were obtained through extraction with 85% ethanol, followed by concentration via rotary evaporation. The resulting residue was suspended in water and sequentially partitioned with ethyl acetate and n-butanol. The organic fractions were further purified by silica gel column chromatography, Sephadex LH-20 gel filtration, MCL gel separation, and semi-preparative RP-HPLC using a CH-based elution system. They exhibit structural diversity through variations in oxidation states and substitution patterns, often containing multiple hydroxyl groups and chiral centers that contribute to their broad biological activities.

**Figure 3:**
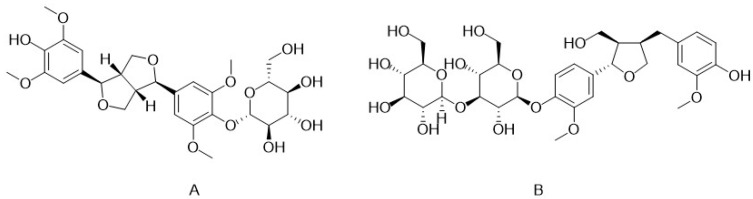
Chemical structures of (+)-syringaresinol-O-β-D-glucopyranoside and (+)-pinoresinol-O-β-D-glucopyranoside in *C. deserticola*. The image illustrates the molecular structures of echinacoside and acteoside. ((**A**), (+)-syringaresinol-O-β-D-glucopyranoside; (**B**), (+)-pinoresinol-O-β-D-glucopyranoside).

In *C. deserticola*, lignans function as phytoestrogens with antioxidant properties mediated by free radical scavenging, although their concentrations are relatively low. To date, three primary lignans have been identified (Table 3). Liriodendrin exhibits hepatoprotective [25,26] and anti-inflammatory effects [27,28], while (+)-syringaresinol-O-β-D-glucopyranoside (Figure 3A) shows hepatoprotective potential [25,26]. (+)-Pinoresinol-O-β-D-glucopyranoside (Figure 3B) induces vasodilation, suggesting potential antihypertensive applications [29]. Additionally, eucommin A, isolated from *C. deserticola*, demonstrates antibacterial activity [30]. These findings highlight the understudied therapeutic value of lignans in this species, emphasizing the need for further phytochemical and pharmacological exploration.

### 3.4. Cistanche Polysaccharides

Cistanche polysaccharides (CPs), composed of glucose, galactose, rhamnose, arabinose, and fructose monomers, are a major class of bioactive components in *C. deserticola*. Their structural heterogeneity, influenced by extraction and purification methods, plays a critical role in determining their biological efficacy (Table 4).

Different extraction protocols yield polysaccharides with distinct bioactivities. For example, thermal-assisted extraction, involving hot water extraction, ethanol precipitation, and column chromatography, produces CP fractions with potent antioxidant activity [31,32]. Cold-water enzymatic extraction, which includes cold-water immersion, hexadecanol precipitation, and ion-exchange chromatography, yields CP fractions that enhance lymphocyte proliferation [33]. Multistep chromatographic purification, involving advanced workflows such as Soxhlet extraction, DEAE cellulose chromatography, and Sephacryl S-300 gel filtration, generates high-purity CPs with enhanced immune regulation capabilities through macrophage activation [7,34]. Ethanol-assisted enzymatic processing, which involves ethanol pretreatment followed by enzymatic hydrolysis and DEAE agarose chromatography, yields CP fractions that modulate gut microbiota composition by promoting probiotic growth [35]. These methodological variations highlight the structure-function relationship of CP polysaccharides, where extraction temperature, solvent selection, and purification techniques collectively influence bioactivity. The antioxidant capacity appears preferentially associated with thermal extraction methods, while immunomodulatory effects correlate with chromatographic refinement processes. Such technological diversity underscores the importance of standardized extraction protocols for reproducible pharmacological applications.

**Table 4:** The influence of different extraction and purification methods on the biological activity of *C. deserticola* polysaccharides.

Solvent Extraction Methods	Monosaccharide Composition	Bioactivity	References
Ethanol soaking, grinding, water dissolution, enzyme treatment, dialysis, concentration, precipitation, washing, drying, and DEAE agarose gel column elution	Glucose and galactose	Promote the health of gut microbiota	[35]
Ultrasonic cellulase-assisted extraction, followed by ethanol precipitation.	Ribose, mannose, glucose, and galactose	Antioxidant	[32]
Hot water extraction, followed by ethanol precipitation and column chromatography for purification.	Glucose, galactose, rhamnose, arabinose, and fructose.	Antioxidant	[31]
Cold water extraction, the hexadecanol precipitation method, and ion exchange chromatography for separation, followed by gel filtration purification.	Arabinose, D-galactose, L-rhamnose, D-galacturonic acid, and trace amounts of D-xylose and D-glucose.	Immune regulatory function	[33]
Soxhlet extraction, air drying, distilled water extraction, concentration dialysis, ethanol precipitation, washing and drying, DEAE cellulose column elution, and Sephacryl S-300 purification.	CDA-1A (homogeneous chitosan) is composed of glucose. CDA-3B (homogeneous polysaccharide) is composed of rhamnose, arabinose, galactose, glucose, and galacturonic acid.	Immune regulatory function	[7]
Ethanol extraction, hot water extraction, filtration centrifugation, ethanol precipitation, protein removal, crude polysaccharide freeze-drying, DEAE column purification, and Sephadry S-300 purification, Sephadex G-25 desalination.	Arabinose, galactose, and glucose, as well as trace amounts of rhamnose and mannose.	Immune regulatory function	[34]

## 4. The Main Pharmacological Functions

Based on research, we have come to understand that *C. deserticola* has various beneficial effects on the human body. Current studies on its functions mainly focus on the following aspects.

### 4.1. Antifatigue Effects of Bioactive Substances

The bioactive compounds of *C. deserticola* enhance energy metabolism, muscle function, and endurance capacity, significantly improving physical performance and quality of life [36,37]. These compounds reduce lactic acid accumulation and oxidative stress, ensuring efficient energy utilization. In animal studies, they have been shown to increase ATP, liver glycogen, and muscle sugar levels while reducing creatinine, uric acid, phosphate, and lactic acid levels, thereby delaying exercise fatigue [38,39]. In vitro experiments have further demonstrated that the active components of *C. deserticola* regulate AMP-activated protein kinase (AMPK) activity, maintaining energy metabolism balance and reducing oxidative stress [40]. The combination of ingredients such as alkaloids, CPs, PhGs, and flavonoids operates in *C. deserticola* to offer the body long-lasting energy, enhance stamina and endurance, and alleviate physical fatigue.

As a kind of Chinese herb widely used in sports and physical labor, the antifatigue mechanisms of *C. deserticola* mainly include promoting energy metabolism, antioxidant effects, promoting blood circulation, and regulating nerve conduction. These mechanisms regulate the physiological activities of the organism, improve metabolic state, reduce oxidative damage, and enhance the oxygen supply capacity of muscles, thereby achieving the antifatigue effect [41]. The effect of the above aspects is interrelated and synergistic, which effectively improves the body’s ability to exercise and resist fatigue.

**Figure 4:**
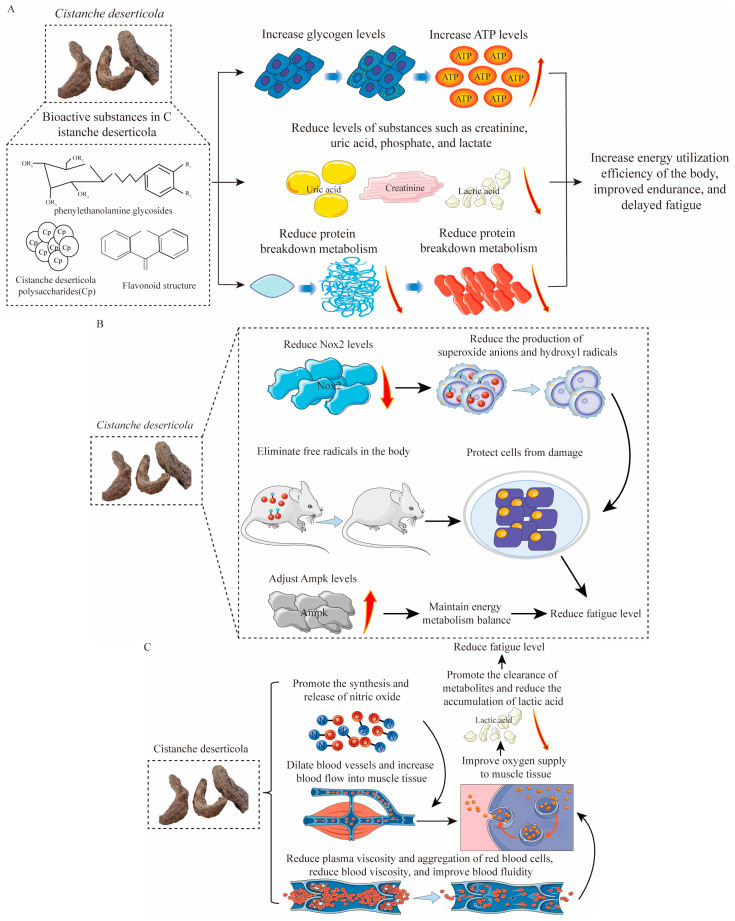

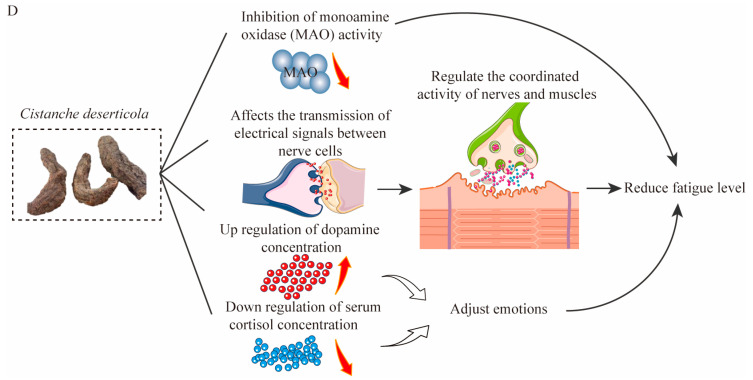
Bioactive compounds from *C. deserticola* exert antifatigue effects through multiple mechanisms. (**A**) enhancing energy metabolism by increasing ATP production and glycogen storage while reducing fatigue markers (creatinine, uric acid, lactic acid) [38,39]; (**B**) mitigating oxidative stress via ROS scavenging (superoxide anions, hydroxyl radicals) and upregulation of antioxidant enzymes (SOD, CAT, GPx) [44,45]; (**C**) improving vascular function through nitric oxide (NO)-mediated enhancement of blood flow and oxygen delivery to muscles [46]; and (**D**) regulating nervous system activity by modulating dopamine/cortisol levels through monoamine oxidase (MAO) inhibition [38].

Firstly, CPs, flavonoids, saponins, PhGs, and other components in *C. deserticola* promote energy metabolism and enhance the efficiency of energy utilization through multiple pathways, alleviating fatigue symptoms [42]. In animal studies, these compounds have been shown to increase ATP, liver glycogen, and muscle sugar levels while reducing creatinine, uric acid, phosphate, and lactic acid levels, thereby delaying the onset of exercise fatigue [38,39]. In addition, active substances in *C. deserticola* can effectively delay and lower the production of lactic acid and decrease the catabolism of proteins, hence decreasing blood urea nitrogen levels and enhancing endurance. Finally, the bioactive compounds of *C. deserticola* can elevate glycogen content, providing more energy supply resources [43]. Therefore, muscle cells are able to obtain energy more effectively to reduce fatigue (Figure 4A).

The bioactive compounds of *C. deserticola* have excellent antioxidant activity, which can effectively eliminate free radicals in the body. Free radicals are very active molecules, and overproduction may lead to structural and functional damage of the cell. In animal studies, active components of *C. deserticola* have been shown to scavenge and eliminate free radicals, reducing cell damage and alleviating fatigue [44,45]. Furthermore, the active constituents of *C. deserticola* have shown great effects in inhibiting fatty acid peroxidation. AMP-activated protein kinase (AMPK) is another key regulatory enzyme of energy metabolism that regulates blood glucose homeostasis and promotes energy metabolism. After heavy exercise, AMPK will be activated, and the components in *C. deserticola* may help to regulate the level of AMPK and keep the balance of energy metabolism [40]. *C. deserticola* can also reduce the level of NADPH oxidase 2 and NOX2 and modulate the inflammatory response through contact catalysis, resulting in limited oxidative stress and reducing a major source of reactive oxygen species. By reducing the NOX2 level, it can reduce the production of superoxide anions and hydroxyl radicals. Therefore, it relieves oxidative damage, including phenomena of hemolysis of erythrocytes and lipid peroxidation of hepatic microsomes induced by ROS, and protects muscular tissue [45]. The active ingredients of *C. deserticola* modulate a series of enzymes’ levels, including phosphokinase and lactate dehydrogenase, while enhancing the levels of catalase, glutathione peroxidase, superoxide dismutase, and two other antioxidant enzymes. Through this balanced regulation, it confers protection upon tissue cells against damage and enhances endurance in exercise. *C. deserticola* further attenuates oxidative damage to cells by capturing free radicals like nitric oxide and malondialdehyde, thereby reducing fatigue [44] (Figure 4B).

The bioactive substances in *C. deserticola* can dilate blood vessels, increasing blood flow to muscle tissues. This improves the supply of oxygen to muscles, facilitating the removal of metabolites and reducing the accumulation of lactic acid, thereby maintaining a good state of oxygen supply and reducing fatigue. In animal studies, these compounds have been shown to enhance the synthesis and release of nitric oxide, improving vascular function and reducing lactic acid accumulation [46]. Additionally, bioactive substances in *C. deserticola* improve endothelial function, prevent blood vessel blockage, reduce the risk of thrombosis, and decrease the likelihood of vascular obstructive diseases. By reducing plasma viscosity and red blood cell aggregation, they improve blood flow, enhancing blood delivery capacity and supplying more oxygen and nutrients to muscle tissues [46]. Thus, the bioactive compounds of *C. deserticola* reduce fatigue through multiple pathways, including enhancing oxygen supply to muscles, promoting metabolite removal, reducing lactic acid accumulation, maintaining vascular patency, and improving blood flow (Figure 4C).

The nervous system is regulated by compounds such as alkaloids, PhGs, and polyphenols. They further modulate the nerve conduction and influence the transmission of electrical signals between the nerve cells, hence helping in the regulation of the coordinated activities of nerves and muscles. As a result, this relieves nervous tension, anxiety, depression, and other feelings that reduce symptoms of fatigue. Moreover, these components can inhibit MAO activity to exert anti-fatigue effects. The active ingredients of *C. deserticola* are known to upregulate the concentrations of dopamine in the brain and down-regulate serum cortisol concentrations. In animal studies, the active ingredients of *C. deserticola* have been shown to upregulate the concentrations of dopamine in the brain and down-regulate serum cortisol concentrations, alleviating stress and anxiety. Dopamine is an excitatory neurotransmitter closely related to feelings of pleasure and fulfillment, and serum cortisol is a hormone associated with stress and anxiety [38]. Therefore, the bioactive components of *C. deserticola* can help alleviate stress and anxiety, improve mood, and further reduce the symptoms of fatigue. Hence, the alkaloids, PhGs, and polyphenols in *C. deserticola* decrease fatigue symptoms by regulating the nervous system, relieving nervous tension and anxiety, decreasing monoamine oxidase activity, antidepressant effects, increasing the concentration of dopamine in the brain, and decreasing the concentration of serum cortisol through combined effects (Figure 4D).

### 4.2. The Immune Regulatory and Mechanisms of Bioactive Substances

The bioactive compounds in *C. deserticola* play a crucial role in immune regulation. Animal studies have demonstrated that these compounds enhance immune responses by promoting the differentiation and activation of immune cells, thereby improving resistance to pathogens and tumor cells [47,48]. In vitro experiments further reveal that *C. deserticola*’s active components activate dendritic cells (DCs) through the TLR4-mediated NF-κB pathway, accelerating T cell proliferation [47]. These findings highlight the potential of *C. deserticola* in boosting immune function and combating infections.

**Figure 5:**
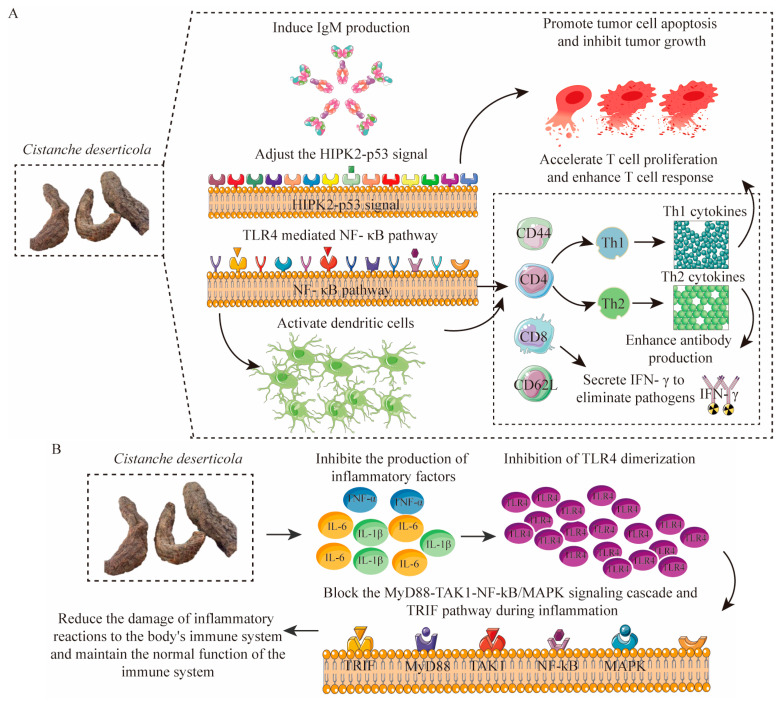
Immunomodulatory mechanisms of bioactive compounds in *C. deserticola*. (**A**) Immune activation through TLR4/NF-κB-mediated dendritic cell maturation and subsequent T-cell proliferation (CD4/CD8/CD44/CD62L), enhancing pathogen defense [47,48,49]; (**B**) Anti-inflammatory action via suppression of MyD88-TAK1-NF-κB/MAPK signaling cascades and downregulation of proinflammatory cytokines (TNF-α, IL-6, IL-1β) [4,33,51,52,53,54], demonstrating dual immunoregulatory capacity.

In animal studies, *C. deserticola* polysaccharides have been shown to significantly enhance CD4, CD8, CD44, and CD62L T cell responses in the spleen and lymph nodes, promoting immune cell proliferation and differentiation [48,49]. Activated CD8 T cells eliminate pathogens by secreting IFN-γ, while proliferating CD4 T cells differentiate into Th1 and Th2 cells. Th1 cells secrete cytokines that stimulate lymphocyte proliferation, and Th2 cells enhance antibody production, further strengthening the immune response. In vitro experiments have confirmed that these bioactive substances improve the morphology of DCs, induce IgM production, and inhibit tumor growth by regulating HIPK2-p53 signaling and promoting apoptosis [47,50] (Figure 5A).

Additionally, the bioactive substances in *C. deserticola* also inhibit the inflammatory response by reducing the release of inflammatory cells and the production of inflammatory factors (TNF-α, IL-6, and IL-1β). In animal studies, these compounds have been shown to alleviate inflammation by blocking the MyD88-TAK1-NF-κB/MAPK signaling cascade and the TRIF pathway, thereby mitigating immune system damage caused by excessive inflammation [4,51]. In vitro experiments have further demonstrated that these substances inhibit TLR4 dimerization, reducing the activation of inflammatory cells and the release of inflammatory mediators [33,52,53,54]. (Figure 5B).

The immunomodulatory effects can enhance the immune response, and anti-inflammatory effects can alleviate the inflammatory response, thus protecting the normal function of the immune system. These findings suggest that *C. deserticola* has dual potential in both the food and biomedical industries, particularly in developing novel immunomodulatory therapies and anti-inflammatory treatments.

### 4.3. Antioxidant Effects

*C. deserticola* is a valuable plant resource rich in bioactive compounds that protect cells and tissues from oxidative damage. Animal studies have shown that these compounds significantly enhance the activity of antioxidant enzymes, including superoxide dismutase (SOD), catalase (CAT), and glutathione peroxidase (GPx), thereby reducing lipid peroxidation and mitigating oxidative stress [44,45]. These findings highlight the potent antioxidant properties of *C. deserticola* in vivo [55,56,57,58].

The bioactive compounds in *C. deserticola* have a highly efficient free radical quenching ability that effectively neutralizes harmful free radicals in the body, such as superoxide anion, hydroxyl radical, and lipoxygen radical. In vitro experiments have confirmed that *C. deserticola* extracts exhibit strong free radical scavenging abilities, including the quenching of DPPH radicals and iron-reducing activity, which are essential for neutralizing harmful free radicals in the body [59]. This ability is essential for preventing free radical-induced oxidative damage, as the accumulation of free radicals can lead to cell membrane damage, protein denaturation, and DNA breaks, which can cause a variety of diseases. These bioactive compounds are also capable of protecting cells from damage caused by oxidative stress. Oxidative stress is a state of imbalance between oxidants and antioxidants that can lead to cell and tissue damage. Additionally, in vitro studies have demonstrated that *C. deserticola*’s active components can inhibit fatty acid peroxidation, a process that leads to cell membrane damage, and enhance the activity of key antioxidant enzymes such as SOD, CAT, and GPx [59,60,61]. The compounds in *C. deserticola* are able to stabilize free radicals by providing electrons or hydrogen atoms, thus reducing the damage caused by oxidative stress. It is also able to induce the activity of a range of antioxidant enzymes, including superoxide dismutase (SOD), catalase (CAT), and glutathione peroxidase (GPx). These enzymes play a key role in scavenging free radicals and reducing peroxides within the cell, thereby inhibiting the formation of lipid peroxidation. Lipid peroxidation is a reaction in which free radicals attack lipid molecules on cell membranes, leading to impaired membrane integrity and function. Enhanced activity of these antioxidant enzymes helps to protect cell membranes from damage to proteins and DNA due to oxidation [62]. The study by Peng et al. [59] further confirmed the antioxidant potential of *C. deserticola*’s bioactive compounds. Their experimental results showed that these compounds not only possess significant oxygen radical scavenging ability but also exhibit iron-reducing activity, with an IC50 value of 11.68 µmol TE/g FW for FRAP (ferric reducing antioxidant power). These metrics are important criteria for evaluating the efficacy of antioxidants, suggesting that the bioactive compounds in *C. deserticola* have a wide range of antioxidant potential.

The antioxidant mechanisms of *C. deserticola*’s bioactive compounds are further supported by their ability to stabilize free radicals and reduce oxidative stress. In animal studies, these compounds have been shown to reduce oxidative stress markers such as nitric oxide and malondialdehyde, thereby alleviating oxidative damage to tissues and enhancing cellular protection [44,45]. In vitro experiments have revealed that *C. deserticola* extracts can scavenge reactive oxygen species (ROS), including superoxide anions and hydroxyl radicals, further reducing oxidative stress and protecting cells from damage [59]. The antioxidant properties of bioactive compounds in *C. deserticola* play a crucial role in immune regulation, anti-fatigue effects, and overall cell and tissue protection. These findings provide a scientific basis for the application of *C. deserticola* in the fields of medicine, nutraceuticals, and food additives.

### 4.4. Antitumor Effects

The bioactive compounds in *C. deserticola* exhibit significant antitumor properties. They primarily exert anticancer effects by inducing programmed cell death in cancer cells and suppressing tumor cell invasiveness and metastasis [63]. Animal studies have further demonstrated that these compounds inhibit tumor cell proliferation and division, effectively slowing tumor growth and spread [39,64,65].

In vitro experiments further support these findings, showing that *C. deserticola* extracts directly suppress tumor cell growth and reduce angiogenesis, thereby limiting nutrient supply to tumors [39,49]. Notably, the total glycosides (TG) from *C. deserticola* were found to significantly reduce the viability of HepG2 cells, with an IC50 value of 35.28 μg/mL [65]. This suggests a potent inhibitory effect on hepatocellular carcinoma cells.

Phenylethanoid glycosides (PhGs), a key class of bioactive compounds in *C. deserticola*, have shown promising antitumor activity. Animal studies indicate that PhGs can inhibit tumor cell growth, suppress angiogenesis, and modulate immune responses against tumors [39,64,65]. In vitro studies further reveal that PhGs not only directly impede tumor cell proliferation but also enhance the sensitivity of cancer cells to chemotherapy, improving treatment outcomes [8,66]. Additionally, PhGs have been found to target and inhibit cancer stem cells—a crucial breakthrough in oncology, as these cells drive tumor recurrence and metastasis due to their self-renewal capacity [8].

### 4.5. Anti-Inflammatory Effects

The bioactive compounds in *C. deserticola* exhibit potent anti-inflammatory effects [67,68]. Their mechanisms of action are complex, involving the inhibition of key inflammatory signaling molecules such as NF-κB. Animal studies have shown that these compounds reduce the production of inflammatory factors, thereby mitigating inflammatory responses.

These bioactive substances are able to reduce DNA damage induced by oxidative stress and UV radiation [69,70]. Oxidative stress is an important factor in the inflammatory response, which leads to cellular damage and tissue destruction. By reducing oxidative stress and DNA damage, these bioactive substances help to protect cells from damage caused by inflammation. During inflammation, the activation and migration of inflammatory cells is a key step in the process leading to tissue damage. The bioactive substances in desert chicory inhibit the activation of these inflammatory cells, such as neutrophils, macrophages, and lymphocytes, while reducing their migration to the site of inflammation. This action contributes to cellular infiltration at the site of inflammation, thereby alleviating and ameliorating inflammatory symptoms. In addition, they work by inhibiting the activation and migration of immune cells. This suggests that these substances not only reduce the production of inflammatory mediators but also regulate the overall response of the immune system to avoid tissue damage caused by over-activation of the immune system.

The bioactive substances in *C. deserticola* collectively exert powerful anti-inflammatory effects through multiple pathways and mechanisms, including influencing inflammatory signaling pathways, reducing DNA damage, inhibiting the production and activity of inflammatory factors, reducing the release of inflammatory mediators, inhibiting the activation and migration of inflammatory cells, and modulating immune responses. These findings provide a scientific basis for the application of *C. deserticola* in the therapeutic prevention of inflammation-related diseases. The bioactive substances in *C. deserticola* can be used to enhance foods with anti-inflammatory effects, thereby reducing the risk of inflammation-related diseases. In addition, they provide a novel source for developing therapeutic agents that could target multiple pathways involved in inflammation. Investigating the potential of integrating these bioactive substances with established treatment modalities can pave the way for novel and more efficacious anti-inflammatory therapies.

## 5. Medicinal and Dietary Applications of *C. deserticola*

*C. deserticola* has significant therapeutic applications in traditional Chinese medicine, particularly in tonifying kidney yang, nourishing essence and blood, and regulating intestinal function. These properties highlight its potential in both the food and biomedical industries. The active compounds in *C. deserticola* can be developed into dietary supplements to support kidney health, sexual function, and overall vitality. Furthermore, they serve as precursors for novel therapeutics targeting kidney-related disorders, endocrine imbalances, and digestive issues.

### 5.1. Medicinal Applications of C. deserticola

In clinical practice of traditional Chinese medicine, *C. deserticola* is commonly applied in the treatment of symptoms such as lower back and knee soreness, fatigue, and sexual dysfunction caused by kidney yang deficiency, as well as symptoms such as constipation, dizziness, tinnitus, and memory loss caused by insufficient essence and blood [49]. In the “Shennong Bencao Jing”, *C. deserticola* is listed as a top-grade medicinal herb, which is recorded to be able to “treat five labor pains and seven injuries, replenish the middle, eliminate cold and heat pain in the penis, nourish the five organs, strengthen yin, benefit essence and qi, and increase offspring”.

#### 5.1.1. Tonifying Kidney Yang

In traditional Chinese medicine, the kidney is considered the “foundation of life”, with kidney yang playing a crucial role in maintaining physiological functions such as warmth in the limbs, mental clarity, normal sexual function, and smooth bowel movements [71]. *C. deserticola*, classified as a “tonifying yang medicine” [39], has a sweet and salty taste, a warm nature, and an affinity for the kidney meridian. According to the theory of meridian attribution, its primary therapeutic effect is the regulation of kidney yang [72]. Kidney yang is one of the most important driving forces for human life activities, and warming kidney yang signifies the promotion of kidney function [73]. Due to the long-term application in clinics, *C. deserticola* has been applied to a variety of symptoms caused by kidney yang deficiency, such as impotence, premature ejaculation, cold pain in the waist and knees, and infertility. As those symptoms improved, it thus proves the effectiveness of tonifying kidney yang by *C. deserticola* [74].

#### 5.1.2. Nourishing Essence and Blood

Nourishing essence and blood involves enhancing the quality and abundance of essential substances to improve physical fitness and resilience. According to the “Shennong Bencao Jing” and other medical texts, *C. deserticola* is known for its warm tonification properties and is particularly effective in treating blood deficiency [2]. Its active ingredients regulate endocrine function, improve hormone balance, and enhance the nourishment of essence and blood. Additionally, *C. deserticola* supports the function of reproductive organs, including the ovaries, testicles, and uterus, demonstrating its synergistic effects in generating and maintaining essence and blood [42].

#### 5.1.3. Regulating Intestinal Function and Promoting Defecation

*C. deserticola* has been traditionally used to regulate intestinal function and promote defecation, attributed to its rich bioactive compounds. Animal studies have shown that these compounds improve intestinal motility and increase stool volume, alleviating constipation [75]. In vitro experiments further demonstrate that *C. deserticola* polysaccharides promote probiotic growth, modulate gut microbiota balance, and enhance nutrient absorption, improving overall digestive health [35]. The bioactive substances in *C. deserticola* also exhibit moisturizing properties, which help soften stool and promote smooth bowel movements. In animal studies, these compounds have been shown to increase gastrointestinal motility and reduce the risk of constipation [75]. In vitro experiments have confirmed that *C. deserticola* extracts can enhance the synthesis of digestive enzymes, further improving intestinal function [35]. Thus, it regulates internal function and encourages defecation.

### 5.2. The Dietary Application of Bioactive Substances in C. deserticola

Research on bioactive substances in *C. deserticola* has primarily focused on CPs, as shown in Table 5. This emphasis is due to the unique properties of CPs, including high bioactivity, digestibility, and absorption, as well as their abundance in *C. deserticola*.

#### 5.2.1. The Application of CPs in the Food Industry

CPs are usually used as thickeners, coagulants, and stabilizers in the food processing industry. When CPs are used, their rheological properties are mainly characterized by changes in viscosity. Changing the viscosity of CP through concentration, temperature, and pH can regulate the consistency and taste of food [76]. In addition, CPs are resistant to acid and high temperature [79] and remain highly stable during the processing and storage of food products [77].

CPs have high viscosity and emulsification properties, which can raise the viscosity of products like jam and beverages well and make their texture more delicate and uniform, thus improving their texture and sensory quality [80]. This helps improve their sensory quality and avoid a nasty experience in taste. CPs also possess potent antioxidant properties that effectively eliminate free radicals and reduce oxidative damage [59,60]. These have particular roles in maintaining the nutritional quality, color, and flavor of food products like jams and beverages [81]. Furthermore, CP thickeners can stabilize and form gels; therefore, they are useful in a number of industrial applications, including food processing. Some of the most promising CPs for application as thickeners and stabilizers in food processing are α-1,4-d-glucan, α-l-arabinyl-3,6-β-d-galactose, pectic CP, and 4-o-methyl-d-glucuronosyl-d-xyloglucan [82]. Moreover, dietary fiber, minerals, and vitamins present in CP can effectively improve the value of nutritional products like jams and beverages. This would, in turn, help satisfy consumer demand for healthier food and enhance market competitiveness for such products.

CPs have a range of health benefits, including immunomodulation, anti-aging, and antifatigue. These CPs also showed the enhancing ability in the absorption of echinacosides, as indicated by Wang et al. [83]. Echinacosides are bioactive compounds that have potential health benefits and a positive effect on gut microbiota [13]. Such CPs may thus be added to functional beverages for added nutritional value and increased marketability in regard to promoting immune health and supplementing energy. This is in agreement with the use of natural products, especially cherries and blueberries, for enhancing the immune system against some chronic diseases in functional beverages [11]. Therefore, adding CPs to functional beverages would be a very promising strategy for promoting human health. The addition of CPs into functional beverages is mainly performed for the high-value utilization in food industries, enhancing the nutritional value and market competitiveness of food products based on CPs. Not only that, but it fully conforms to the current concern of consumers about health and nutrition but also to the current development trend of the food industry toward natural, organic, and functional food.

As consumer awareness of health and nutrition continues to rise, the utilization of CP (chitosan phosphate) in the food industry is poised to see significantly broader application. It can be used around a number of foods, such as bakery foods, sugar confectionery, and health food. It can be used as a component in health-functional foods and as an additive to functional health food products. CP extract and many other kinds of deep-processed products are possible to produce, which will satisfy the diversified demands of consumers. The deep-processed product application prospect also includes CP extract. If technology further advances and research deepens, we will have an even deeper processing of a greater variety of products using the special effect of CPs. Further development of CPs into functional food products implies enormous potential for these traditional herbs in making a new range of health-promoting functional foods, beverages, sweets, etc. for improving health and well-being. Therefore, CPs have shown great potential for application in the food industry.

#### 5.2.2. Utilization as a Drug Stabilizer

CP is commonly used in capsules, tablets, and injections in the pharmaceutical industry. It turned out from the investigation results that different microstructures of CP are established in different formulations. For example, in the formulation for capsules, it will be granular, which will sustain medication stability and discharge. In injections, the microstructure of CP is at the fiber form, which can enhance the stability and durability of the drug [9,32,78]. Moreover, CP exhibits certain biological activities. Plenty of research proves that CP can improve human health by modulating their immune system and antioxidant capacity. Bioactivities of CPs are closely correlated with their microstructure, and different microstructures would make different interactions and effects with biomolecules. Thus, studying and optimizing the microstructure of CP can lead to enhanced efficacy and safety of CP in drug development and clinical application.

## 6. Conclusions and Future Perspectives

Recent research on *C. deserticola* has significantly advanced our understanding of its nutritional and health benefits. Pharmacological studies have demonstrated its diverse medicinal properties, including hormone regulation, immunomodulation, and antioxidant, anti-inflammatory, and neuroprotective activities. Additionally, *C. deserticola* has been shown to enhance immunity, reduce fatigue, combat aging, and improve learning and memory. These effects are attributed to its rich array of bioactive compounds, such as phenylethanol glycosides (PhGs), polysaccharides, iridoids, and lignans, which exhibit potent therapeutic potential. Future research should focus on optimizing extraction and purification methods to ensure the stability and efficacy of these compounds in food and pharmaceutical applications. Innovative products targeting specific health issues, such as osteoporosis, vascular dementia, and inflammation, hold great promise.

However, research on *C. deserticola* faces significant challenges. The complexity of its chemical composition, particularly the diversity and concentration of its active ingredients, requires advanced analytical techniques for comprehensive characterization. Quality control and standardization are critical to ensure the consistency and safety of *C. deserticola*-based products. Additionally, the increasing scarcity of wild *C. deserticola* resources necessitates the development of sustainable harvesting and cultivation techniques to meet growing demand while preserving ecological balance.

Interdisciplinary collaboration among botany, pharmacology, analytical chemistry, and molecular biology is essential to address these challenges. Effective communication and coordination among researchers, industry stakeholders, and regulatory bodies are crucial to ensure compliance with relevant regulations and promote the responsible development of *C. deserticola*-based products. Furthermore, while preliminary research has demonstrated its pharmacological potential, clinical validation remains insufficient. Rigorous clinical trials are needed to confirm the efficacy and safety of *C. deserticola* in humans, particularly for its applications in treating chronic diseases and improving overall health.

Current patents related to *C. deserticola* primarily focus on optimizing processing, extraction, and purification techniques to enhance the efficiency and quality of its active components. Innovations such as low-temperature drying, ultrasound-assisted extraction, and chromatographic purification have significantly improved the utilization of its bioactive compounds. These advancements not only reduce production costs but also support large-scale industrial applications. Moreover, patent protection enables companies to establish technological barriers, enhancing their market competitiveness. With the growing demand for natural products and the adoption of sustainable practices, *C. deserticola* is poised to play a pivotal role in the health industry, offering opportunities for both ecological conservation and economic benefits.

International collaboration and knowledge exchange are particularly important in advancing *C. deserticola* research. By leveraging global expertise and resources, researchers can accelerate the discovery of novel bioactive compounds, elucidate their mechanisms of action, and develop innovative applications in medicine, nutraceuticals, and functional foods. This collaborative approach will provide a robust scientific foundation for the development of new drugs, dietary supplements, and health products, ultimately contributing to improved human well-being.

In conclusion, *C. deserticola* represents a valuable resource with immense potential in both traditional and modern medicine. By addressing current challenges and fostering interdisciplinary and international collaboration, we can unlock the full potential of this remarkable plant, paving the way for its sustainable utilization and widespread application in health and wellness.

## Figures and Tables

**Table 1:** The pharmacological functions of bioactive substances in *C. deserticola.*

Compounds	Active Action	References
Phenylethanol glycosides	Anti-inflammatory, antioxidant, regulates osteoblast differentiation and mineralization, anti-hypoxic myocardial injury, improves renal injury, anti-osteoclastogenesis, anti-ischemia, hepatoprotective, anti-viral, improves glucose tolerance, regulates blood glucose, neuroprotective, inhibits postprandial glucose elevation, improves glucose tolerance, inhibits vasodilatation, suppresses appetite, antidiabetic, anticancer, antidiabetic vascular complication	[8,9,10]
Iridoid	Anti-inflammatory, antioxidant, antibacterial, antithrombotic, antidepressant, antidiabetic, anti-kidney injury, hepatoprotective, estrogenomimetic, inhibits postprandial glucose elevation, neuroprotective	[11]
Lignans	Antivasorelaxant, hepatoprotective, anti-inflammatory, antimicrobial, anti-kidney injury, neuroprotective, antioxidant, inhibit postprandial glucose elevation	[2,12]
Cistanche polysaccharides	Anti-fatigue, laxative, energizing, anti-viral, anti-inflammatory	[13]

**Table 2:** The main composition of components in PhGs from *C. deserticola.*

Compounds	Active Action	References
Echinacoside	Anti-cerebral ischemia, liver protection, and anti-inflammatory	[14,15]
Acteoside	Hypoxia-resistant myocardial injury and anti-vascular dementia	[18]
Tubuloside A	Anti-aging and liver protection	[16]
Tubuloside B	Protect the nerves and protect the liver	[17]
2′-Acetylacteoside	Antibone resorption cell generation	[20]
Isoacteoside	Improve kidney injury	[19]

**Table 3:** The main composition of components in lignans from *C. deserticola.*

Compounds	Bioactivity	References
(+)-Pinoresinol	Vasodilator	[29]
(+)-Pinoresinol-O-β-D-glucopyranoside	Liver protection	[25]
(+)-Syringaresinol-O-β-D-glucopyranoside	Liver protection	[26]
Liriodendrin	Anti-inflammatory and liver protection	[27,28]
Eucommin A	Antibacterial	[30]

**Table 5:** The application mechanism of CPs in different fields.

Application Area	Application Methods	Mechanism of Action	Application Prospect	References
Food industry	Thickener, coagulant, stabilizer	Adjust viscosity, improve product stability, and improve taste	Can be widely used in fields such as pastries, candies, and health foods to meet consumer needs	[76,77]
Medical field	Capsules, tablets, injections	Adjust microstructure and enhance drug stability	Can be used for the development of various drug formulations, improving efficacy and safety	[78]
Functional foods	Extracts	Increase the nutritional value of products	Improve product competitiveness and adapt to consumer demand for healthy food	[76]
Deeply processed products	Extracts, facial mask, health products	Develop more products with special functions	Meet the needs of different demand groups	[31]

## Data Availability

No data were used for the research described in the article.

## Abbreviations

*C. deserticola*, *Cistanche deserticola* Y. C. Ma; PhGs, phenylethanol glycosides; CP, *C. deserticola* polysaccharide; ORAC, oxygen radical absorbance capacity; DPPH, 2,2-diphenyl-1-pyridine hydrazine; FRAP, ferric reducing antioxidant property; AMPK, AMP-dependent protein kinase; NOX2, NADPH oxidase 2; ROS, reactive oxygen species; MAO, monoamine oxidase; DC, dendritic cell.
